# Precision Nursing Device Management and Artificial Intelligence-Integrated Early Warning Systems for Central Nervous System Infections in the Neurocritical Care Unit: A Scoping Review

**DOI:** 10.7759/cureus.111026

**Published:** 2026-06-17

**Authors:** Youtian Zhou, Hong Xie, Meirong Hu, Gaoquan Luo

**Affiliations:** 1 Department of Infectious Diseases, General Hospital of the Southern Theater Command, Guangzhou, CHN; 2 Department of Emergency Medicine, General Hospital of the Southern Theater Command, Guangzhou, CHN; 3 Department of Neurosurgery, General Hospital of the Southern Theater Command, Guangzhou, CHN

**Keywords:** artificial intelligence, central nervous system infections, critical care nursing, device management, early warning systems, neurocritical care

## Abstract

Central nervous system (CNS) infections and clinically overlapping neuroinflammatory conditions in the neurocritical care unit (neuro-ICU) are associated with profound mortality and prolonged clinical burdens. Invasive neuromonitoring devices, particularly external ventricular drains (EVDs), significantly increase the risk of infection, while delayed clinical manifestations often hinder early recognition and intervention. This scoping review aimed to systematically map the current landscape of precision nursing device management and artificial intelligence (AI)-integrated early warning systems (EWS) for CNS infections in the neuro-ICU, rather than to definitively evaluate their clinical effectiveness. Guided by the Preferred Reporting Items for Systematic Reviews and Meta-Analyses extension for Scoping Reviews (PRISMA-ScR) framework, a systematic search of four major databases was conducted. Eligibility criteria included studies focusing on adult patients in the neuro-ICU. A rigorous multi-round filtration process was applied. A total of 24 eligible studies, encompassing longitudinal nursing management cohorts, quality improvement protocols, and machine learning predictive models, were meticulously selected for data extraction and narrative synthesis. The systematic optimization of EVD care bundles, specifically the reduction of routine cerebrospinal fluid sampling and the implementation of closed needleless systems, significantly decreased device-related infection rates, occasionally achieving zero infections under optimal interdisciplinary rounding conditions. Furthermore, AI-driven EWS algorithms (e.g., Random Forest, XGBoost {Seattle, WA: University of Washington}) and non-invasive intracranial pressure (ICP) waveform clustering demonstrated exceptional prognostic accuracy. These advanced models successfully predicted ventriculitis up to 24 h prior to positive bacterial cultures. The management of severe complications, such as paroxysmal sympathetic hyperactivity, remains heavily reliant on continuous evidence-based nursing vigilance. The integration of AI-driven predictive modeling with standardized, precision nursing bundles represents a paradigm shift from reactive treatment to proactive prevention in neurocritical care. Translating these technologies into bedside clinical decision support systems will be pivotal in optimizing individualized patient outcomes and redefining neuro-ICU nursing standards.

## Introduction and background

Central nervous system (CNS) infections, encompassing meningitis, infectious encephalitis, and healthcare-associated ventriculitis, as well as clinically overlapping conditions such as autoimmune encephalitis that present with similar neurological deterioration and require comparable ICU nursing vigilance, represent catastrophic emergencies in the neurocritical care unit (neuro-ICU). The epidemiological burden of these infections is profound. Recent multi-center data demonstrate an exceedingly high in-hospital mortality rate of 15.3% among ICU patients with CNS infections, while adult meningitis cohorts experience ICU admission rates approaching 15% due to severe complications such as seizures and cerebral edema [1,2]. Furthermore, the routine deployment of invasive neuromonitoring devices, particularly external ventricular drains (EVDs), significantly amplifies the susceptibility to healthcare-associated infections. For instance, the incidence of ventriculostomy-associated infections (VAI) in critically ill patients can reach alarming rates of up to 47%, profoundly prolonging mechanical ventilation duration and overall ICU length of stay [3].

Within this highly complex clinical environment, critical care nurses serve as the vanguard in infection prevention and recognition of clinical deterioration. Stringent precision nursing device management is operationalized through meticulous adherence to evidence-based standardized care bundles, which serve as the cornerstone of infection prevention. Authoritative clinical performance measures strongly advocate for standardized EVD insertion and maintenance protocols as indispensable quality indicators in the neuro-ICU [4]. Longitudinal and quality improvement studies have consistently shown that systematically optimizing EVD care bundles, such as restricting routine cerebrospinal fluid (CSF) sampling and implementing interdisciplinary rounding models, can safely and substantially reduce device-related infection rates, sometimes driving them to near-zero levels [5,6].

Despite the proven efficacy of standardized nursing interventions, the early identification of insidious CNS infections remains a formidable clinical challenge. Traditional infection-specific clinical judgment heavily relies on overt physiological manifestations, which are often delayed or masked by critical illness and sedation. Consequently, there is an urgent need for a paradigm shift towards advanced neuromonitoring and the integration of artificial intelligence (AI). Non-invasive dynamic indicators, such as intracranial pressure (ICP) waveform morphological clustering, have emerged as potent biomarkers capable of predicting ventriculitis days before bacterial cultures yield positive results [7]. Concurrently, machine learning (ML) architectures, including Random Forest and XGBoost algorithms (Seattle, WA: University of Washington), are being aggressively deployed to pinpoint cryptic risk factors and prognosticate patient outcomes with unprecedented accuracy [8,9].

While existing reviews typically examine infection prevention or AI monitoring in isolation, this scoping review distinctively maps the synergistic integration of precision nursing management and advanced predictive technologies for CNS infections in the neuro-ICU. Therefore, this scoping review aimed to systematically map and synthesize the current landscape of nursing device management, multidisciplinary collaboration protocols, and AI-integrated early warning strategies. By delineating these critical domains, this review seeks to provide a robust, evidence-based foundation for optimizing neurocritical nursing practice and guiding future clinical decision support system development.

In this review, early warning systems (EWS) refer to any systematic approach, whether based on static risk factors or dynamic physiological monitoring, that provides clinicians with actionable information regarding the risk of incident CNS infection or clinical deterioration, with a lead time ranging from hours (e.g., ICP waveform changes) to days (e.g., ML-based admission risk stratification).

## Review

Methods

Study Design

To systematically map the current landscape of nursing management and early warning strategies for central nervous system (CNS) infections in the neurocritical care unit (neuro-ICU), a scoping review was conducted. The methodology of this review strictly adhered to the Preferred Reporting Items for Systematic Reviews and Meta-Analyses extension for Scoping Reviews (PRISMA-ScR) guidelines [10]. This design was selected because it enables the comprehensive integration of diverse study designs, including observational cohorts, quality improvement protocols, and machine-learning predictive models, to clarify complex nursing concepts and identify knowledge gaps in clinical practice. The completed PRISMA-ScR checklist is provided in the appendix.

Search Strategy

An initial comprehensive literature search was conducted in major electronic databases (up to May 24, 2026) to identify studies on CNS infections, EVD management, and neurocritical care nursing, yielding an initial pool of 170 references. Furthermore, to ensure the review captured the most cutting-edge advancements and epidemiological contexts, a supplementary targeted search (via citation chaining and specific AI-related MeSH terms) was subsequently performed to identify recent literature focusing specifically on artificial intelligence predictive models and neuro-ICU disease burdens.

To ensure methodological transparency and reproducibility, the full electronic search strategy utilized for the databases (e.g., PubMed) is provided as follows: ("Central Nervous System Infections" OR "Meningitis" OR "Encephalitis" OR "Cerebral Ventriculitis" OR "Ventriculitis" OR "brain abscess") AND ("Intensive Care Units" OR "Intensive Care" OR "Critical Care" OR "Neurocritical Care" OR "Neuro-ICU") AND ("Nursing" OR "Nursing Care" OR "Critical Care Nursing" OR "nursing management" OR "Early Warning" OR "Clinical Deterioration" OR "nurse intuition" OR "nurse worry" OR "Early Warning Score" OR "clinical alarm"). All database searches were executed on May 24, 2026, and limited to publications from the past 10 years (2016-2026).

Inclusion and Exclusion Criteria

To guarantee the clinical relevance and professional depth of this review, rigorous inclusion and exclusion criteria were established. First, studies were included if they focused on adult patients admitted to the neuro-ICU. Second, the research was required to involve specific CNS infections, including healthcare-associated ventriculitis, post-craniotomy meningitis, or severe encephalitis. Finally, eligible articles must have primarily investigated nursing interventions, medical device management (e.g., EVD care bundles), or early warning systems (EWS).

Conversely, several exclusion criteria were applied to refine the selection. Studies were excluded if they focused on neonatal or pediatric populations. Additionally, literature that solely investigated pharmacological or pathophysiological mechanisms without actionable nursing perspectives was omitted. Furthermore, the review excluded purely macro-epidemiological surveys lacking specific ICU scenarios, as well as isolated case reports lacking predictive value or replicable nursing protocols.

Study Selection and Data Extraction

**Figure 1:**
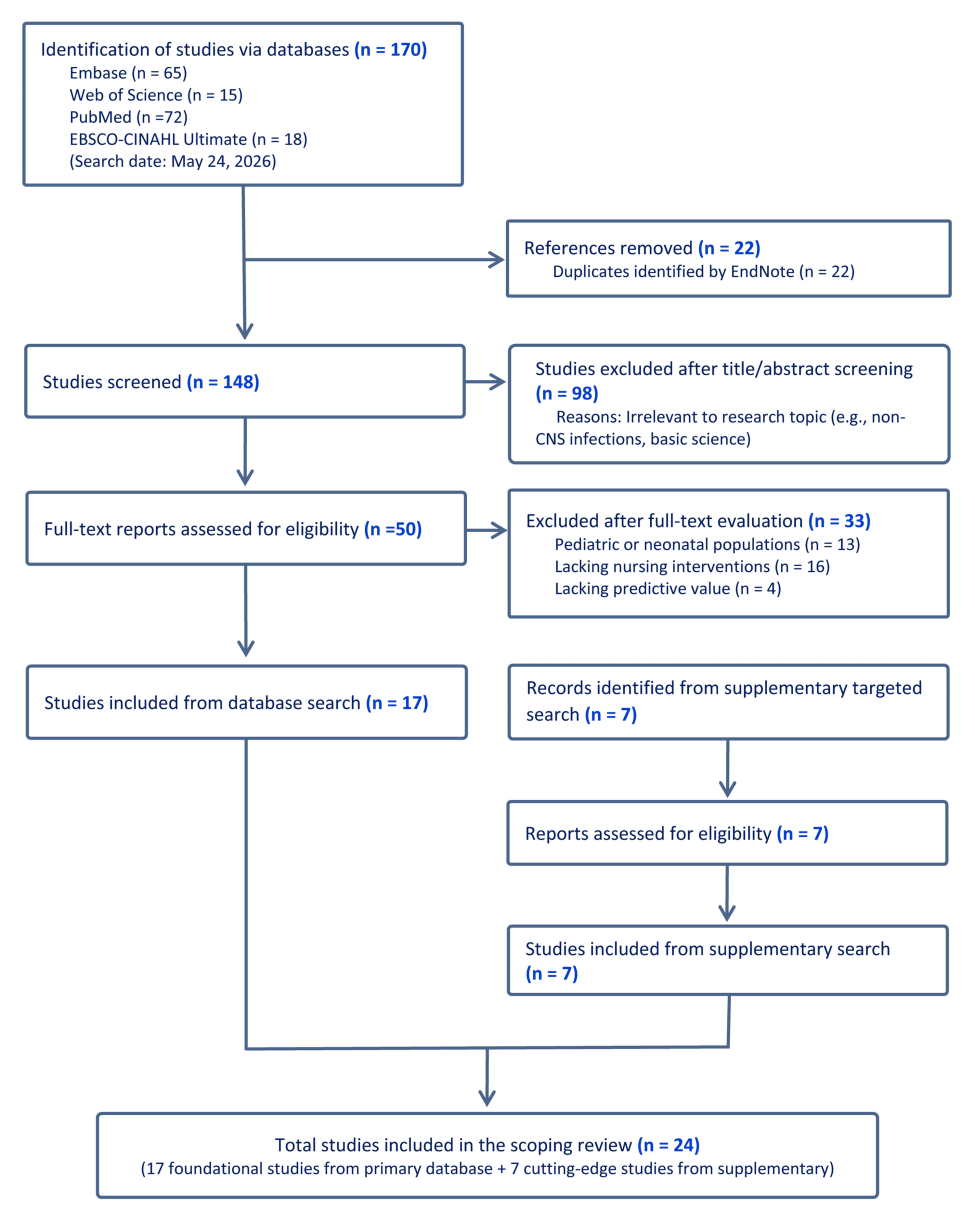
PRISMA-ScR flow diagram illustrating the dual-pathway study selection process. This diagram details the systematic literature search and rigorous multi-round filtration protocol guided by the PRISMA-ScR framework. An initial database search (PubMed, Embase, Web of Science, and CINAHL) yielded 170 records, while a supplementary targeted search (via citation chaining and AI-related MeSH terms) identified an additional seven records. Following the removal of 22 duplicates and strict full-text eligibility screening, a total of 24 high-quality core studies, encompassing foundational nursing management and cutting-edge AI predictive models, were ultimately included in the scoping review. PRISMA-ScR: Preferred Reporting Items for Systematic Reviews and Meta-Analyses extension for Scoping Reviews

The study selection process utilized a dual-pathway approach. For the initial database search (n=170), following the removal of 22 duplicates using EndNote 2025 (Build 19000) (Philadelphia, PA: Clarivate), 148 records underwent a rigorous two-stage screening protocol (title/abstract screening followed by full-text evaluation). After title, abstract, and full-text screening, 17 foundational core studies were retained. Concurrently, the supplementary targeted search identified an additional seven highly relevant studies focusing on machine learning algorithms and macro-epidemiology. Consequently, a total of 24 eligible studies (17 from the primary search and seven from the supplementary search) were ultimately included in this scoping review (Figure 1).

Data extraction was systematically performed independently by two reviewers using a standardized charting form. Any discrepancies were resolved through consensus. The extracted variables included the first author, publication year, study design, sample size, core nursing interventions or EWS indicators, and key clinical outcomes. Consistent with the PRISMA-ScR framework, a formal critical appraisal or risk-of-bias assessment was not conducted, as the primary objective of this scoping review was to map the breadth of available evidence rather than to evaluate the methodological quality of individual studies.

Results

Literature Selection, Patient Demographics, and Epidemiological Burden

A total of 24 highly relevant studies were ultimately included in this scoping review, encompassing 17 core studies on nursing management and early warning indicators, alongside seven recent studies focusing on cutting-edge predictive models and epidemiology (Figure 1). The selected literature consists of prospective and retrospective cohorts, quality improvement longitudinal studies, machine-learning (ML) predictive modeling research, and consensus clinical practice guidelines (Table 1).

**Table 1:** Characteristics and core extracted data of the included studies (n=24). The table summarizes the 24 core studies divided into the following four distinct domains: nursing device management, AI-driven predictive models, clinical recognition with complication management, and epidemiological burden with guidelines. EWS: early warning systems; EVD: external ventricular drain; CHG: chlorhexidine gluconate; ML: machine learning; RF: Random Forest; SHAP: Shapley Additive Explanations; AUC: area under the curve; AUROC: area under the receiver operating characteristic curve; NMDAR: N-methyl-D-aspartate receptor; ICP: intracranial pressure; GCS: Glasgow Coma Scale; VAI: ventriculostomy-associated infections; PSH: paroxysmal sympathetic hyperactivity; NANDA-NIC-NOC: North American Nursing Diagnosis Association, Nursing Interventions Classification, and Nursing Outcomes Classification XGBoost (Seattle, WA: University of Washington)

Studies	Study design and sample size	Core nursing interventions/EWS indicators	Key clinical outcomes and findings
Part 1: nursing device management and infection prevention bundles			
Salman et al. (2026) [11]	Case series, n=5	Needleless CSF sampling: using a needleless extension (three-way valve) to maintain sterility during active CSF exchange	Demonstrated a safe approach for reducing infection risk; described as a painless innovation during CSF sampling and medication delivery
Harvey et al. (2025) [6]	Quality improvement longitudinal	Interdisciplinary EVD rounding: regular rounding model involving neurosurgery, nursing, and infection prevention teams	EVD-related ventriculitis rates sustained reduction (from 24.7 to 2.97 per 1000 line days), achieving zero infections in the final 12 months
Huang et al. (2024) [12]	Retrospective cohort	Standardized EVD care bundles: inclusion of chlorhexidine gluconate (CHG) bathing and strict insertion checklists	Significant reduction in EVD infection rates from 3.6% to 1.0% (p=0.03)
Caroti et al. (2024) [13]	Narrative review	Safe EVD management protocols for infection prevention and control	Emphasized the critical role of nursing checklists and strict aseptic techniques in drain management
Walek et al. (2021) [5]	Longitudinal study 12-year experience	Unbundling routine care: decreasing routine CSF sampling frequency (to three times weekly) and using alcoholic chlorhexidine	Safely and significantly decreased EVD-related infections over 12 years (regression coefficient {β}=3.91; p=0.011)
Whyte et al. (2020) [14]	Retrospective cohort	Impact of EVD bundle combined with limited duration antibiotic prophylaxis	Significantly decreased drain-related infections and minimized antibiotic resistance risks
Rivas-Rodriguez et al. (2016) [15]	Retrospective study	Risk assessment associated with suboptimal EVD catheter care and insertion point management	Suboptimal catheter care was associated with a significantly higher risk of ventriculitis (OR: 3.8, 95% CI: 1.1-13.9)
Part 2: AI-driven predictive models and EWS			
Guo et al. (2025) [9]	Retrospective cohort (machine learning), n=121	Predicting autoimmune encephalitis prognosis using Random Forest (RF) and XGBoost with SHAP analysis	RF achieved highest accuracy (AUC: 0.950). SHAP identified infection and CSF monocyte percentage as core predictive indicators
Wang et al. (2025) [16]	Retrospective cohort (machine learning), n=140	ML and SHAP value integration for anti-NMDAR encephalitis prognosis. Model deployed as a Web app	RF model achieved R²=0.706. ICU admission identified as the paramount predictive feature (SHAP value=1.65)
Savin et al. (2018) [8]	Retrospective cohort (machine learning)	Tree-based ML approaches (XGBoost/RF) to identify risk factors for healthcare-associated ventriculitis/meningitis	EVD presence, craniotomy, and CSF leak pinpointed as the foremost non-linear predictors of infection
Lu et al. (2023) [17]	Retrospective cohort	Prediction model (nomogram) for CNS infections after severe traumatic brain injury and craniotomy	Model incorporated CSF leak and albumin, achieving outstanding predictive performance (AUC: 0.962)
Megjhani et al. (2022) [18]	Retrospective cohort	Machine learning representations of dynamic ICP waveform morphology	Automated continuous monitoring achieved an AUROC of 0.70 for predicting ventriculitis
Megjhani et al. (2021) [7]	Retrospective cohort	Automated clustering analysis of non-invasive ICP waveforms	Morphological shifts (single-peak/artifactual) accurately predicted ventriculitis 1 day prior to positive culture (p<0.0001)
Chen et al. (2016) [19]	Retrospective case-control, n=4392	Early warning risk factors associated with post-craniotomy meningitis	EVD placement >72 h, prolonged surgical duration, and CSF leak validated as critical EWS risk markers
Part 3: clinical recognition and complication management			
Mower (2017) [20]	Case study/narrative	Frontline nursing assessments (e.g., GCS, fever with lethargy) for early recognition of encephalitis	Emphasized that early recognition by nurses prevents clinical misdiagnosis and devastating neuropsychological impairments
Wang et al. (2021) [21]	Retrospective observational	Nursing surveillance for paroxysmal sympathetic hyperactivity (PSH) in severe anti-NMDAR encephalitis	PSH occurred in 50% of cases (tachycardia/hyperthermia), significantly prolonging mechanical ventilation and NICU stay
Song et al. (2025) [22]	Prospective observational	Strict nursing protocols for intraventricular/intrathecal polymyxin B injections for multidrug-resistant CNS infections	Achieved a high bacterial clearance rate of 81.5% through 15-min slow injection and 60-min drain occlusion
Vicente and Segovia (2016) [23]	Case report	Continuity of nursing care for severe anti-NMDAR encephalitis using the NANDA-NIC-NOC framework	Prolonged nursing plans addressing self-care deficits and caregiver strain proved crucial for recovery until discharge
Part 4: epidemiological burden and guidelines			
Andrade et al. (2024) [1]	Multi-center retrospective, n=451	Macro-epidemiological survey of CNS infections in the ICU	Revealed an in-hospital mortality rate of 15.3%. Mechanical ventilation ≥10 days was an independent predictor (OR=6.1)
Turon et al. (2025) [3]	Prospective observational, n=271	Ventriculitis incidence in patients with aneurysmal subarachnoid hemorrhage requiring EVD	Incidence of ventriculostomy-associated infections (VAI) reached 47%, with a mean diagnosis time of 4.4 days post-insertion
Waqar et al. (2022) [2]	Retrospective cohort	Risk factors for intensive care unit admission and mortality among adult meningitis patients	ICU admission rate was 14.8%, driven by risk factors such as diabetes and seizure onset. Mortality rate was 10.5%
Livesay et al. (2020) [4]	Consensus guidelines	Clinical performance measures for neurocritical care by the Neurocritical Care Society (NCS)	Advocated formalized EVD insertion bundles as core quality indicators to yield infection rates as low as 1%
Cook et al. (2020) [24]	Consensus guidelines	Guidelines for the acute treatment of cerebral edema in neurocritical care patients	Recommended strict nursing surveillance for adverse effects when administering adjunctive corticosteroids or hyperosmolar agents
Ogbebor et al. (2023) [25]	Narrative review	Multidisciplinary collaboration and management of neurological emergencies in the ICU	Highlighted the necessity of prompt nursing response and dynamic monitoring in complex CNS deterioration scenarios

The epidemiological burden of central nervous system (CNS) infections in the neuro-ICU is substantial. Recent multi-center data revealed a high in-hospital mortality rate of 15.3% for ICU patients with CNS infections, with mechanical ventilation for ≥10 days serving as a significant independent predictor of mortality [1]. Furthermore, adult meningitis patients face a 14.8% ICU admission rate, driven by risk factors such as diabetes and seizure onset [2]. Device-related infections also pose a severe challenge; for instance, the incidence of ventriculostomy-associated infections (VAI) in patients with aneurysmal subarachnoid hemorrhage requiring external ventricular drains (EVD) reached 47%, with a mean diagnosis time of 4.4 days post-insertion [3].

Nursing Device Management and Infection Control Bundles

Strict nursing protocols and device management are fundamental to preventing healthcare-associated CNS infections. Longitudinal evidence over a 12-year period confirmed that systematically unbundling routine EVD care, specifically by reducing cerebrospinal fluid (CSF) sampling frequency to three times weekly and utilizing alcoholic chlorhexidine preparations, can safely and significantly decrease EVD-related infections (regression coefficient {β}=3.91; p=0.011) [5]. The implementation of standardized EVD care bundles, including chlorhexidine gluconate bathing, significantly reduced EVD infection rates from 3.6% to 1.0% (p=0.03) [12]. Another quality improvement study reported a sustained reduction in EVD-related ventriculitis rates from 24.7 to 2.97 per 1000 EVD line days, achieving zero infections in the final 12 months under optimal interdisciplinary rounding conditions [6]. These findings, however, originate from individual studies and should be interpreted cautiously, as they may not be broadly generalizable across institutions and patient populations. Other narrative reviews and retrospective cohorts further corroborated that multi-item nursing bundles, including insertion checklists and the avoidance of routine daily CSF sampling, significantly decrease drain-related infections and antibiotic resistance [13,14]. In terms of device innovation, the use of a needleless extension system with a three-way valve was demonstrated to maintain strict sterility during active CSF exchange, providing a safe and painless approach to CSF sampling and medication delivery [11]. Suboptimal catheter care, including prolonged drain permanence and improper insertion-site care, was initially associated with a significantly higher risk of ventriculitis (OR: 3.8; 95% CI: 1.1-13.9), underscoring the need for rigorous nurse training [15].

Advanced Early Warning Systems (EWS) and Artificial Intelligence Integration

The integration of advanced neuromonitoring and artificial intelligence (AI) has significantly transformed early warning capabilities in the neuro-ICU. Traditional EWS parameters, such as EVD placement >72 h, prolonged surgical duration, and CSF leak, have been validated as critical risk markers for post-craniotomy meningitis [19]. To enhance predictive accuracy, nomogram models incorporating nursing-monitored variables (e.g., CSF sampling frequency, CSF leak, and serum albumin levels) yielded outstanding performance for predicting CNS infections after severe traumatic brain injury, achieving an area under the curve (AUC) of 0.962 in the training cohort and 0.942 in the validation cohort [17].

The application of ML algorithms represents a major paradigm shift in critical care prognostication. Tree-based ML approaches, including XGBoost and Random Forest, have effectively managed non-linear interactions to pinpoint EVD, craniotomy, and CSF leak as the foremost predictors of healthcare-associated ventriculitis and meningitis [8]. In the context of autoimmune encephalitis, Random Forest algorithms achieved an AUC of 0.950 in the training cohort and 0.920 in the independent validation set for prognostic prediction; coupled with Shapley Additive Explanations (SHAP) analysis, infection status and CSF monocyte percentage were identified as core indicators [9]. Another ML-SHAP-integrated model for anti-N-methyl-D-aspartate receptor (NMDAR) encephalitis (R²=0.706) highlighted ICU admission as the most predictive feature (SHAP value=1.65) [16]. Regarding non-invasive continuous monitoring, automated clustering analysis of non-invasive intracranial pressure (ICP) waveforms proved that a morphological shift to single-peak or artifactual distributions could accurately predict ventriculitis up to one day prior to a positive bacterial culture (p<0.0001) [7], achieving an area under the receiver operating characteristic curve (AUROC) of 0.70 via machine learning representations (Table 2) [18].

**Table 2:** Summary of AI-driven early warning systems (EWS) and predictive models. This table highlights the specific algorithm architectures, target clinical outcomes, and core predictive features extracted from the machine learning studies included in this scoping review. Only the top predictive features identified by SHAP or model importance are listed; refer to original studies for complete feature sets. EWS: early warning systems; ML: machine learning; XGBoost: Extreme Gradient Boosting; SHAP: Shapley Additive Explanations; NMDAR: N-methyl-D-aspartate receptor; EVD: external ventricular drain; ICP: intracranial pressure; AUC: area under the curve; AUROC: area under the receiver operating characteristic curve; MAE: mean absolute error XGBoost (Seattle, WA: University of Washington)

Studies	Model type/algorithm	Target disease/outcome	Core predictive features (EWS)	Predictive performance
Guo et al. (2025) [9]	Machine learning (Random Forest, XGBoost)+SHAP	Prognosis of antibody-positive autoimmune encephalitis	Infection status, CSF monocyte percentage, prealbumin	Random Forest achieved highest accuracy: AUC=0.950 (training), AUC=0.920 (independent validation set)
Wang et al. (2025) [16]	Machine learning (Random Forest)+SHAP	Prognosis of anti-NMDAR encephalitis	ICU admission (SHAP=1.65), memory decline, uric acid	R²=0.706, MAE=2.512 (five-fold cross-validation)
Savin et al. (2018) [8]	Tree-based machine learning (XGBoost, Random Forest)	Healthcare-associated ventriculitis and meningitis	EVD presence, craniotomy, superficial infection, CSF leak	Successfully managed non-linear clinical interactions (10-fold CV)
Lu et al. (2023) [17]	Nomogram prediction model	CNS infection after severe traumatic brain injury	CSF sampling frequency, CSF leak, serum albumin levels	AUC=0.962 (training), AUC=0.942 (validation)
Megjhani et al. (2022) [18]	Machine learning (waveform morphology representation)	Dynamic prediction of ventriculitis via ICP	Continuous non-invasive ICP pulses	AUROC=0.70 for predicting ventriculitis (500-iteration bootstrapping with 20% hold-out)
Megjhani et al. (2021) [7]	Automated clustering analysis	Early warning of EVD-associated ventriculitis	Morphological shifts from triphasic to single-peak/artifactual waveforms	Predicted ventriculitis up to 24 h prior to positive culture (p<0.0001; change point analysis without train/test split)

Clinical Recognition and Multidisciplinary Complications Management

Early recognition of encephalitis in acute settings relies heavily on frontline nursing assessments to prevent devastating neuropsychological impairments and mortality resulting from clinical misdiagnosis [20]. Multidisciplinary collaboration is explicitly endorsed by authoritative guidelines, which mandate standardized insertion bundles to achieve EVD-related infection rates as low as 1% [4] and recommend strict nursing surveillance for adverse effects when administering adjunctive corticosteroids for cerebral edema [24,25]. For highly complex cases, such as severe anti-NMDAR encephalitis, prolonged North American Nursing Diagnosis Association, Nursing Interventions Classification, and Nursing Outcomes Classification (NANDA-NIC-NOC) nursing plans addressing self-care deficits and caregiver strain are crucial for continuity of care until discharge [23]. Moreover, nurses must be highly vigilant for severe autonomic complications; paroxysmal sympathetic hyperactivity (PSH) occurs in 50% of severe anti-NMDAR encephalitis cases, presenting with tachycardia and hyperthermia, which significantly prolongs mechanical ventilation and NICU stay [21]. When multidrug-resistant CNS infections occur, strict nursing administration protocols for intraventricular or intrathecal polymyxin B injections (e.g., slow injection over 15 min followed by 60 min of drain occlusion) have demonstrated high efficacy, achieving a bacterial clearance rate of 81.5% [22].

Discussion

Paradigm Shift in Nursing Device Management: From Routine to Precision Care

**Figure 2:**
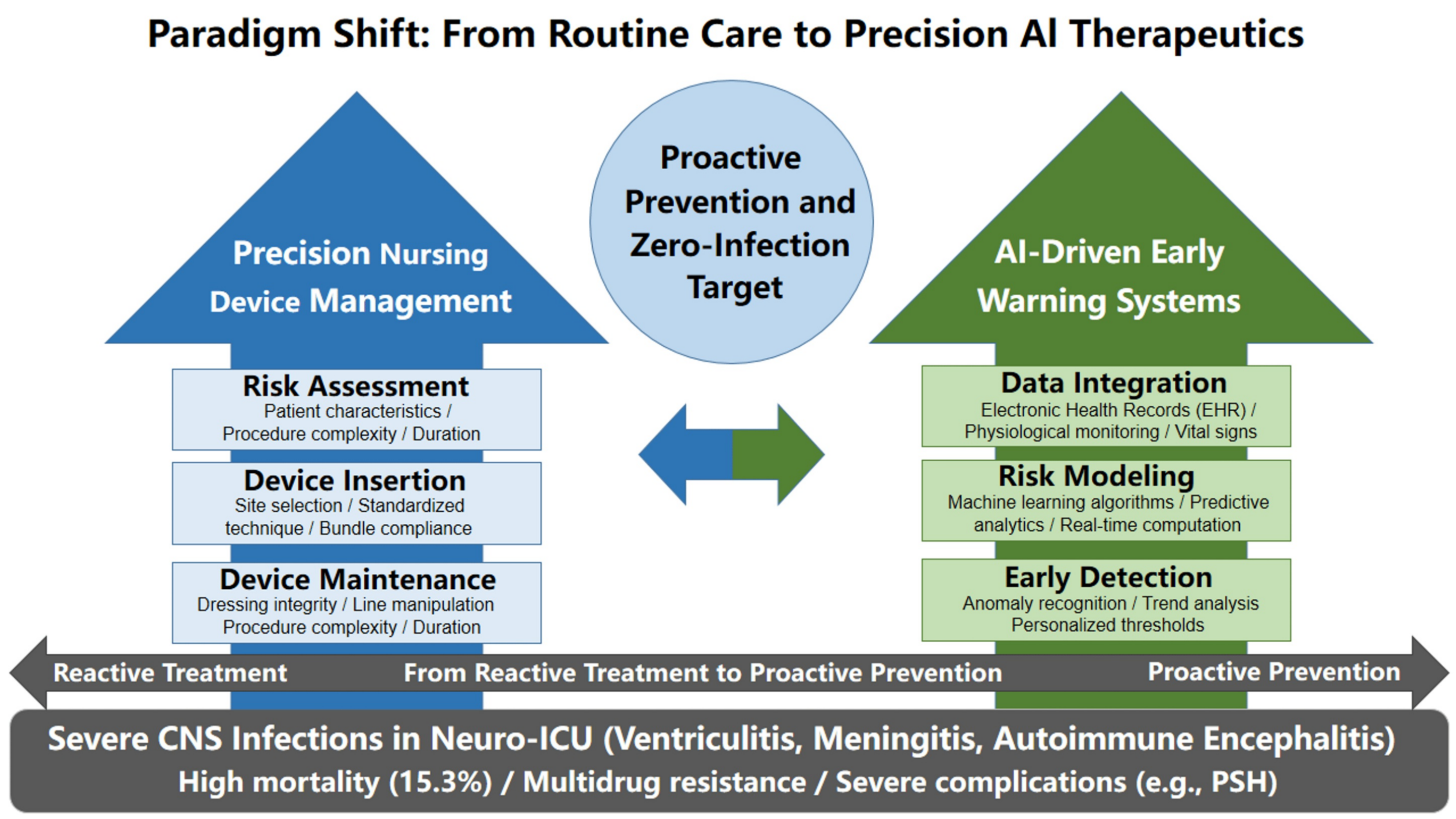
Conceptual framework of the paradigm shift in the management of central nervous system (CNS) infections within the neuro-ICU. The model demonstrates the synergistic integration of two core pillars: precision nursing device management (left) and AI-driven early warning systems (right). Precision management encompasses EVD bundle optimization, technological innovations (e.g., closed needleless extension systems), and interdisciplinary clinical protocols. Concurrently, AI-driven systems leverage machine learning algorithms (e.g., Random Forest, XGBoost), SHAP explainability, and dynamic neuromonitoring to achieve early risk detection. The bidirectional interaction between these approaches drives the transition from routine, reactive care to proactive prevention, ultimately advancing toward a zero-infection target. This figure represents a conceptual model synthesizing the review findings, rather than direct quantitative evidence derived from the included studies. Neuro-ICU: neurocritical care unit; EVD: external ventricular drain; SHAP: Shapley Additive Explanations; ICP: intracranial pressure; CHG: chlorhexidine gluconate; PSH: paroxysmal sympathetic hyperactivity This image was created by the authors of this study using Microsoft PowerPoint software. XGBoost (Seattle, WA: University of Washington); Microsoft PowerPoint (Redmond, WA: Microsoft Corp.)

The findings of this scoping review highlight a pivotal paradigm shift in the nursing management of external ventricular drains (EVDs) and invasive devices within the neuro-ICU (Figure 2).

Historically, routine cerebrospinal fluid (CSF) sampling and prophylactic interventions were standard practice; however, longitudinal evidence increasingly advocates for a targeted, "less is more" approach. The systematic "unbundling" of routine care, specifically by restricting CSF sampling solely to clinically indicated scenarios and strictly adhering to alcoholic chlorhexidine preparations, has been shown to drastically reduce EVD-related infections over a 12-year period [1]. This transition is further augmented by localized, interdisciplinary EVD rounding models, which have demonstrated the capability to drive infection rates down to absolute zero [6]. Furthermore, the adoption of closed, needleless extension systems during active CSF exchange represents a critical technological innovation. Such systems maintain absolute sterility while facilitating painless medication delivery and sampling, effectively minimizing exogenous pathogen exposure [11]. These findings strongly echo the authoritative consensus that institutional compliance with formalized, evidence-based insertion bundles is non-negotiable for achieving minimal infection rates in neurocritical care [4].

Evolution of Early Warning Systems: The Synergy of AI and Neuromonitoring

A core elevation of modern neurocritical care lies in the integration of artificial intelligence (AI) and machine learning (ML) into early warning systems (EWS). Traditional infection-specific clinical judgment heavily relied on delayed, subjective clinical manifestations. In contrast, current ML architectures, such as Random Forest and XGBoost, exhibit exceptional diagnostic accuracy in identifying insidious risk factors for healthcare-associated ventriculitis and meningitis [8]. More importantly, the application of Shapley Additive Explanations (SHAP) values has bridged the "black box" gap inherent in AI models. By offering highly interpretable prognostic features (e.g., ICU admission status, prealbumin levels, and CSF monocyte percentages), these models empower frontline nurses to make real-time, evidence-based decisions for patients with autoimmune encephalitis [9,16]. Beyond biochemical markers, automated clustering analysis of non-invasive intracranial pressure (ICP) waveform morphology represents a groundbreaking dynamic biomarker. While invasive EVD monitoring remains essential for therapeutic drainage, the addition of non-invasive ICP waveform analysis provides a complementary, continuous surveillance tool without additional infection risk, making it particularly valuable for early detection, either before or in the absence of invasive devices. The ability to detect morphological shifts (e.g., transition from triphasic to single-peak or artifactual waveforms) up to 24 h prior to positive bacterial cultures equips critical care nurses with a crucial temporal window for pre-emptive intervention, fundamentally shifting nursing practice from reactive treatment to proactive prevention [7]. The ultimate output of AI-driven EWS is a clinically actionable risk alert (e.g., elevated probability of ventriculitis within the next 24 h), which directly informs a precision clinical decision (e.g., targeted CSF sampling or prophylactic antibiotic review), closing the loop from prediction to intervention. However, it must be noted that exceptionally high predictive accuracy in some machine learning models raises concerns regarding potential overfitting. Extensive external validation is imperative to ensure model generalizability before widespread clinical implementation.

Mastering Complex Complications and Multidisciplinary Therapeutics

The complexity of CNS infections in the neuro-ICU necessitates highly specialized nursing protocols, particularly when managing severe complications and advanced pharmacotherapeutics. For instance, severe anti-NMDAR encephalitis is frequently complicated by paroxysmal sympathetic hyperactivity (PSH), a condition characterized by profound autonomic instability (e.g., hyperthermia, tachycardia) that occurs in half of the patients and significantly prolongs mechanical ventilation and ICU length of stay [21]. Frontline nurses must possess the clinical acumen to differentiate these autonomic storms from primary septic deterioration in order to initiate appropriate symptom-management protocols promptly. Simultaneously, in the era of multidrug-resistant pathogens, the administration of intraventricular or intrathecal antibiotics (such as Polymyxin B) relies entirely on rigorous nursing execution. Adherence to precise administration protocols, such as slow injection over 15 min followed by strict 60-min drain occlusion, is paramount for achieving high bacterial clearance (up to 81.5%) while mitigating neurotoxicity [22]. Ultimately, navigating these acute phases must be seamlessly connected to long-term continuity of care. Utilizing standardized taxonomies such as the NANDA-NIC-NOC framework ensures that the patient's holistic needs, including alleviating caregiver role strain, are addressed comprehensively from the acute ICU phase through discharge.

Limitations and Future Directions

While this scoping review comprehensively maps the current landscape of nursing management for CNS infections, certain limitations must be acknowledged. First, consistent with scoping review methodology, there is an absence of formal quality assessment or risk-of-bias evaluation, which restricts the ability to assess the strength of the evidence presented. Second, there is a potential for publication bias. Third, there is a limited number of explicitly AI-focused studies. Finally, there is a heavy reliance on observational evidence. Despite the eligibility of the included studies, the inherent heterogeneity in ML algorithms and variations in clinical care bundles across different institutions limit the immediate standardization of a universal protocol. Furthermore, none of the included AI models have undergone prospective bedside implementation or randomized controlled trials to assess their real-world impact on clinical outcomes. Critical barriers to clinical translation, such as real-time data integration, alert fatigue, and nurse-machine interface design, remain largely unaddressed. Therefore, future research must move beyond retrospective validation to pragmatic clinical trials of AI-driven clinical decision support systems (CDSS) within routine neuro-ICU nursing workflows.

## Conclusions

In conclusion, this scoping review underscores the critical role of precision nursing management and advanced early warning systems (EWS) in mitigating the profound clinical burden of central nervous system infections within the neuro-ICU. The systematic implementation of standardized device care bundles, particularly the judicious optimization of cerebrospinal fluid sampling and strict adherence to interdisciplinary protocols, has proven highly effective in minimizing healthcare-associated ventriculitis. Furthermore, addressing highly complex conditions, including multidrug-resistant infections and severe autonomic complications, strictly requires comprehensive, evidence-based nursing vigilance and multidisciplinary collaboration.

Concurrently, the integration of artificial intelligence and machine learning into neuromonitoring marks a transformative leap from reactive treatment to proactive prevention. Interpretable machine learning algorithms and non-invasive dynamic indicators equip frontline nurses with crucial lead time to intercept clinical deterioration. Moving forward, future research must prioritize translating these AI-driven predictive models into bedside clinical decision support systems (CDSS). Empowering neurocritical care nurses with real-time, algorithmic insights has the potential to optimize individualized patient care and enhance clinical outcomes. However, there is currently insufficient prospective clinical evidence to conclude that they will definitively redefine standards of care; rigorous prospective validation remains essential.

## Appendices

**Table 3:** Preferred Reporting Items for Systematic Reviews and Meta-Analyses extension for Scoping Reviews (PRISMA-ScR) checklist. *Where sources of evidence (see second footnote) are compiled from, such as bibliographic databases, social media platforms, and websites. **A more inclusive/heterogeneous term used to account for the different types of evidence or data sources (e.g., quantitative and/or qualitative research, expert opinion, and policy documents) that may be eligible in a scoping review as opposed to only studies. This is not to be confused with information sources (see first footnote). ***The frameworks by Arksey and O’Malley (6) and Levac and colleagues (7) and the JBI guidance (4, 5) refer to the process of data extraction in a scoping review as data charting. ****The process of systematically examining research evidence to assess its validity, results, and relevance before using it to inform a decision. This term is used for items 12 and 19 instead of "risk of bias" (which is more applicable to systematic reviews of interventions) to include and acknowledge the various sources of evidence that may be used in a scoping review (e.g., quantitative and/or qualitative research, expert opinion, and policy document). JBI: Joanna Briggs Institute

Section	Item	PRISMA-ScR checklist item	Reported on page
Title			
Title	1	Identify the report as a scoping review	Title page (identified as scoping review)
Abstract			
Structured summary	2	Provide a structured summary that includes (as applicable): background, objectives, eligibility criteria, sources of evidence, charting methods, results, and conclusions that relate to the review questions and objectives	Abstract section
Introduction			
Rationale	3	Describe the rationale for the review in the context of what is already known. Explain why the review questions/objectives lend themselves to a scoping review approach	Introduction (paragraphs 1-3)
Objectives	4	Provide an explicit statement of the questions and objectives being addressed with reference to their key elements (e.g., population or participants, concepts, and context) or other relevant key elements used to conceptualize the review questions and/or objectives	Introduction (paragraph 4)
Methods			
Protocol and registration	5	Indicate whether a review protocol exists; state if and where it can be accessed (e.g., a Web address); and if available, provide registration information, including the registration number	N/A (not registered)
Eligibility criteria	6	Specify characteristics of the sources of evidence used as eligibility criteria (e.g., years considered, language, and publication status), and provide a rationale	Methods, inclusion and exclusion criteria subhead
Information sources*	7	Describe all information sources in the search (e.g., databases with dates of coverage and contact with authors to identify additional sources), as well as the date the most recent search was executed	Methods, search strategy subhead, and Figure 1
Search	8	Present the full electronic search strategy for at least 1 database, including any limits used, such that it could be repeated	Methods, search strategy subhead
Selection of sources of evidence**	9	State the process for selecting sources of evidence (i.e., screening and eligibility) included in the scoping review	Methods, study selection and data extraction subhead
Data charting process***	10	Describe the methods of charting data from the included sources of evidence (e.g., calibrated forms or forms that have been tested by the team before their use, and whether data charting was done independently or in duplicate) and any processes for obtaining and confirming data from investigators	Methods, study selection and data extraction subhead
Data items	11	List and define all variables for which data were sought and any assumptions and simplifications made	Methods, study selection, and data extraction subhead (variables extracted: author, year, design, sample size, EWS indicators, outcomes)
Critical appraisal of individual sources of evidence****	12	If done, provide a rationale for conducting a critical appraisal of included sources of evidence; describe the methods used and how this information was used in any data synthesis (if appropriate)	N/A (not required/performed for this scoping review)
Synthesis of results	13	Describe the methods of handling and summarizing the data that were charted	Methods, study selection and data extraction subhead, and narrative synthesis mapped in results
Results			
Selection of sources of evidence	14	Give numbers of sources of evidence screened, assessed for eligibility, and included in the review, with reasons for exclusions at each stage, ideally using a flow diagram	Methods, study selection and data extraction subhead; Results, study selection subhead, and Figure 1
Characteristics of sources of evidence	15	For each source of evidence, present characteristics for which data were charted and provide the citations	Results, study selection subhead, Tables 1, 2
Critical appraisal within sources of evidence	16	If done, present data on critical appraisal of included sources of evidence (see item 12)	N/A (scoping review)
Results of individual sources of evidence	17	For each included source of evidence, present the relevant data that were charted that relate to the review questions and objectives	Results, relevant subheads, Tables 1, 2
Synthesis of results	18	Summarize and/or present the charting results as they relate to the review questions and objectives	Results, relevant subheads
Discussion			
Summary of evidence	19	Summarize the main results (including an overview of concepts, themes, and types of evidence available), link to the review questions and objectives, and consider the relevance to key groups	Discussion, relevant subheads
Limitations	20	Discuss the limitations of the scoping review process	Discussion, limitations, and future directions subhead
Conclusions	21	Provide a general interpretation of the results with respect to the review questions and objectives, as well as potential implications and/or next steps	Conclusion paragraph
Funding			
Funding	22	Describe sources of funding for the included sources of evidence, as well as sources of funding for the scoping review. Describe the role of the funders of the scoping review	Title page/declarations
